# Segmentation of the Breast Skin and Its Influence in the Simulation of the Breast Compression during an X-Ray Mammography

**DOI:** 10.1100/2012/876489

**Published:** 2012-05-02

**Authors:** J. A. Solves Llorens, M. J. Rupérez, C. Monserrat, E. Feliu, M. García, M. Lloret

**Affiliations:** ^1^Instituto Interuniversitario de Investigación en Bioingeniería y Tecnología Orientada al Ser Humano/LabHuman, Universitat Politècnica de València, Camino de Vera s/n, 46022 Valencia, Spain; ^2^Unidad RM, Hospital Clínica Benidorm, Avenida Alfonso Puchades 8, 03501 Benidorm, Spain; ^3^Servicio de Radiodiagnóstico de Adultos, Hospital Universitari y Politècnic La Fe de Valencia, Bulevar Sur, 46026 Valencia, Spain

## Abstract

A novel method of skin segmentation is presented aimed to obtain as many pixels belonging to the real skin as possible. This method is validated by experts in radiology. In addition, a biomechanical model of the breast, which considers the skin segmented in this way, is constructed to study the influence of considering real skin in the simulation of the breast compression during an X-ray mammography. The reaction forces of the plates are obtained and compared with the reaction forces obtained using classical methods that model the skin as a 2D membranes that cover all the breast. The results of this work show that, in most of the cases, the method of skin segmentation is accurate and that real skin should be considered in the simulation of the breast compression during the X-ray mammographies.

## 1. Introduction

Currently, the most common imaging modalities used to diagnose breast cancer is the X-ray mammography, magnetic resonance imaging (MRI), and ultrasound. Each imaging modality displays the information of the breast tissues differently. Researchers have found that a combination of these imaging modalities leads to a more effective diagnosis and management of the breast cancer [1]. However, an accurate fusion of the information from different imaging modalities is not trivial. This is not only due to differences in the physics of each imaging modality, but also because the internal tissues deform differently as a result of the differing loading conditions applied during breast imaging [2]. For example, the breast is compressed between two plates during X-ray mammography whereas the breast is typically pendant during MR imaging.

In the last decade, research has been focused on developing algorithms to fuse the information of the different imaging modalities, which take into account the loading conditions of the breast during the imaging procedure. Thus, including realistic models of the breast deformation in these algorithms has gained significant interest. A great number of groups have proposed different biomechanical models of the breast for different applications. Some groups have used homogeneous models [3–7]. However, there are other groups that, in searching for more realistic and accurate models, have proposed heterogeneous models, that is, models that take into account the three tissues of the breast. For example, Ruiter et al. [8] tested different heterogeneous model combinations (from linear to exponential) to register MRI with X-ray mammograms, Kellner et al. [9] used a linear elastic model for each tissue to simulate the mechanical compression of the breast, del Palomar et al. [10] used a neo-Hookean model for the fat and glandular tissues and a Polynomial model for the skin tissue, aimed to study the effect of gravity for surgical planning, and Tanner et al. [11] applied a heterogeneous model consisting of linear elastic and exponential material models also for the register between MRI and X-ray mammograms. Nevertheless, although these models have considered the real fat and glandular tissues obtained, for example, from an MRI or CT, none of them have considered the real skin, but the skin has been approximated as a membrane of constant thickness which covers all the breast.

To the authors' knowledge, there are no studies that compare significant differences when an approximate skin or a more accurate one is used in the simulation of the mammographic compression. While the distribution of fat and dense tissues have been many times represented using the finite element methods, modeling the influence of the skin in the breast biomechanical models still requires further investigation. The simplest options have involved modeling the skin layer as additional 3D elements that surround the internal tissue elements or coupling 2D membrane (skin) elements to 3D fat/dense elements. However, the skin obtained with these approximations is a constant surface which does not take into account issues like widened skin due to suspicious masses near skin area [12, 13] or a differentiated nipple region. Therefore, there is a need to develop a segmentation method for the skin able to remove the real skin (i.e., the external tissue surrounding the breast) from the rest of the breast. This method is aimed to let a correct segmentation of the breast in order to construct an accurate biomechanical model that can be used in the different simulations of the different imaging modalities.

In this paper, a novel method for a more accurate skin segmentation is presented. This method will allow further studies about the impact of accurate skin in biomechanical models, dense/fatty tissue segmentation without skin interference, or accurate density breast analysis. In addition, a biomechanical model of the breast, which considers the skin segmented in this way, is constructed to study the influence of considering real skin in the simulation of the breast compression during an X-ray mammography.

## 2. Materials and Methods

### 2.1. Skin Segmentation

**Figure 1:**
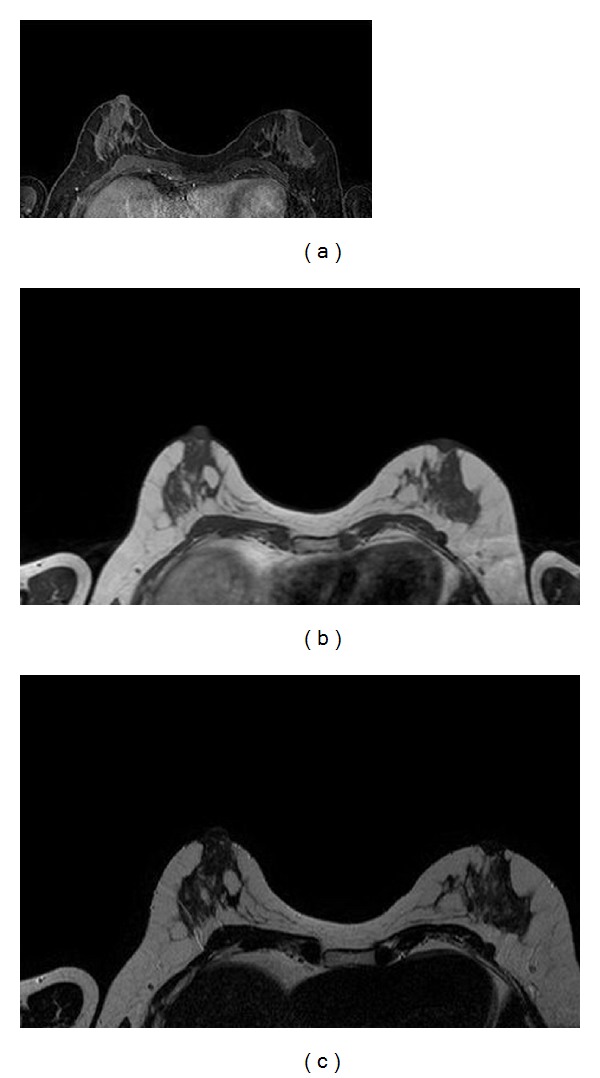
Different MRI (T1 with fat suppression, T1, T2) with skin intensity level similar to dense tissue intensity level.

Breast MRI provides 3D images to cover the whole breast and shows a good contrast between the two major tissues (fatty and dense). Various studies [14–16] have used this difference in signal intensities to separate both tissues. However, there are some limitations. One problem in the breast MRI segmentation is the confusion between skin tissue and dense tissue due to the signal intensities of both tissues are similar and can be easily misclassified if they are not handled properly (Figure 1). The impact of skin in breast MRI segmentation has been shortly investigated, and it is shown that a correct skin removal provides a better density measurement [17].

For this study, a total of 20 cases were segmented. For every case, left and right breasts were segmented separately, with a broad spectrum of breast densities and breast sizes, excluding any case with significative abnormal masses because they were out of the objectives of this work. Fifteen different breasts were selected randomly for the evaluation by three experts. MRIs were acquired using a Philips Achieva 1.5T scanner. Analyzed images were obtained with an axial T2 TSE configuration with TR = 5000 ms, TE = 120 ms, flip angle = 90°, slice thickness was of 2 mm, image height was 512 pixels, image width was 512 pixels, and the matrix size was 448 × 512. T2 sequence displayed 80 slices with high gray level values (white) for fatty tissues and low gray level values (black) for dense and skin tissues due to the time employed by hydrogen protons to offset after radio frequency pulse. This sequence was used due to its ability to display breast structures clearly (with less internal noise than other sequences), its number of slices, and because it does not employ any signal suppression to mask tissues. MRIs were presented in a digital Imaging and communication in medicine format (DICOM). Each DICOM image contained 80 slices with a separation of 2 mm and 65535 different gray levels.

**Figure 2:**
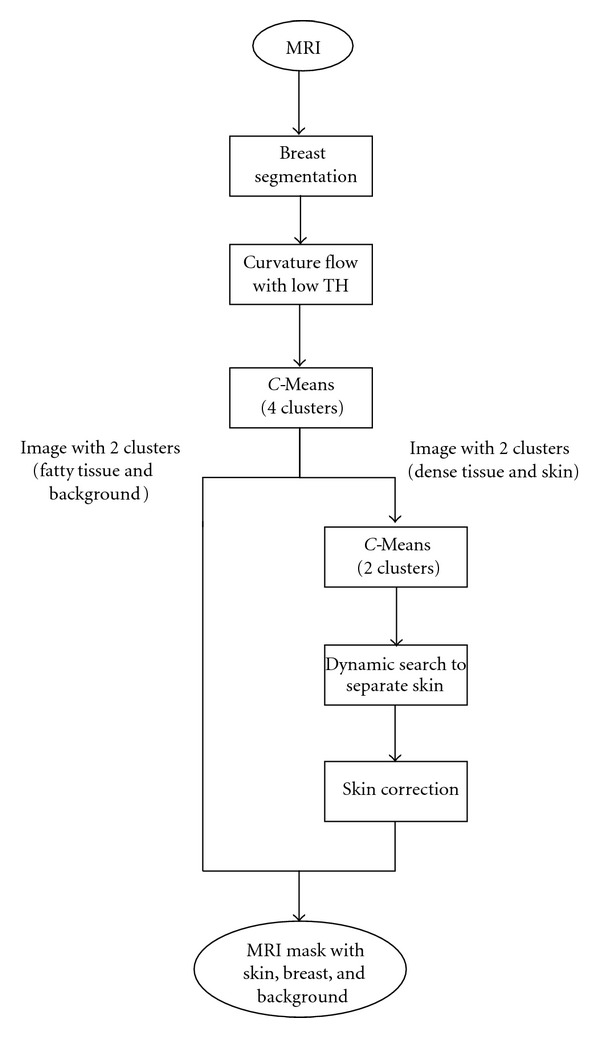
Skin segmentation process.

Skin segmentation process was formed by a sequence of stages (Figure 2). In a first step, right and left breasts are separated from axial zone and one from each other. An easy and fast method to separate breast from axial zone is used to search lowest middle point between both breasts (from all slices) with a high gray level variation and to use that point as a frontier. With the axial zone segmented, each breast can be obtained deleting the right or left side of the image to get left or right breast, respectively.

With both breasts separated one from each other and separated of the axial zone, its differentiation from background is handled. T2 MRIs have random noise surrounding breast that may vary with each case: patient's breathing, noise added by the machine, and so forth. In order to “standardize” each MRI, a curvature flow filter [18, 19] was implemented and applied to images. Curvature flow performs edge-preserving smoothing (similar as an anisotropic diffusion would do) with a level set formulation [20–22]. The intensity contours of an image are used as level sets, where each intensity pixel value forms one level set; the resulting level set function evolves under the control of a diffusion equation where the speed is proportional to the contour's curvature as (1) shows:

(1) It=k|∇I|.

In this equation, *k* is the curvature of the contour, *It* is the level set, and ∇*I* is the image gradient. With this filter, noise artifacts disappear quickly while large scale interfaces evolve slowly. This slowly evolution allows preservation of boundaries. Typical value for time step is 0.0625 in 3D images and, for the number of iterations, is 10 (obtained experimentally, above this number, there was a higher finalization time but no significant improvement). More iterations would result in further smoothing (affecting breast boundaries) and would increase linearly the computing time. After applying curvature filter, a low threshold operation is needed: with the curvature filter, background artifacts are deleted or grouped in low-level gray values around the breast. A low threshold operation with a value obtained experimentally (a value equivalent to 6 if rescaled to a 255 histogram values) managed those little groups.

With noise reduced as explained before, a cluster analysis was performed to the filtered MRI with *C*-means. *C*-means is an unsupervised classification method that has been widely used in breast segmentation [16, 23, 24]. A partition with 4 clusters was enough to divide the MRI in four parts: the darker one, that belongs to background and darkest dense parts of breast, a brighter part, that takes fatty tissues and two clusters that mixe skin and internal breast dense tissues. Only pixels that belonged to the mixed clusters were obtained from original MRI and reclassified with a new 2 clusters *C*-means (which did not use pixels from background or fatty clusters). This new classification was able to reveal a skin layer that surrounded the breast but still classified some dense tissue in that cluster. Adding those two new clusters to the older ones, an image was obtained with a differentiated background.

**Figure 3:**
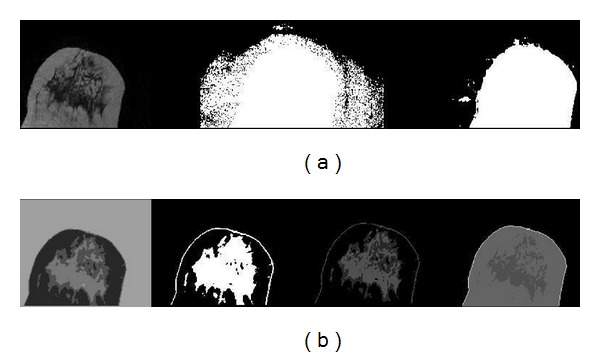
From left to right (a): original MRI slice; pixels with gray value higher than 0; pixels with gray value higher than 0 after curvature flow filter and low threshold. From left to right (b): *C*-means with 4 clusters; clusters with skin and dense tissue; new *C*-means with 2 clusters; final skin classification (white pixel values).

Once the biggest object that does not belong to background (the breast) is chosen and an open-close operation is applied (to smooth breast borders), it is possible to separate skin from dense tissue using a dynamic search from the breast boundary to breast interior with a limit of 3 mm in nipple region (this is the region where skin and dense tissue may be in contact) as suggested in bibliography [17]. If there were isolated pixels classified as dense in contact with skin, they were passed to the skin. With dense tissue isolated from skin, it was easy to change every pixel between breast skin limits to another class value (Figure 3).

**Figure 4:**
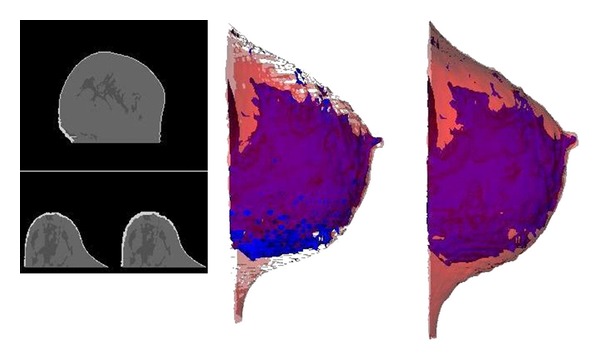
From left to right: one thickness skin artifact (up) and skin growing effect after skin pixel addition (down); skin with holes; reconstructed skin.

Final segmented image needed some improvements. Skin-dense tissue separation explained above can leave skin artifacts inside the breast, with a thickness value of one pixel or dense tissue artifacts with a thickness value of one pixel attached to the skin. Those artifacts are removed with a smoothness iterative filter applied inside the breast that searches skin pixels or dense pixels (in contact with skin) with 3 of its 4 connected neighbor pixels with a value that does not belong to skin or dense cluster, respectively. Skin segmentation method described in this work is able to segment skin in 2D slices, but 3D nature of DICOM images leaves spaces between slices. Those interslice millimeters may produce zones in a 3D reconstruction without skin, leaving internal breast tissues in contact with exterior. To solve this problem, an iterative 3D search adds skin pixels to internal tissues in contact with background (Figure 4). The final pixels labeled as skin were used as a mask to delete skin from original MRI, letting it ready for studies and analysis without skin interaction.

### 2.2. Fat and Dense Tissue Segmentation

With the skin removed, a partition clustering algorithm (*C*-Means) that searches for an optimal partition of the data into 2 clusters was used. The objective of the algorithm is to minimize the sum of squared errors (SSE) of the partition into *C* clusters (2), where *x* ∈ *X* is a data element and *mC* is the cluster *C* mean:

(2) $$\underset{i}{\sum }\underset{j}{\sum }={||x_{i}-mCj||}^{2}.$$

The data is distributed randomly into each cluster, and then the algorithm chooses an optimal partition minimizing the cluster SSE, thus, proving the impact in the SSE formula by moving each data element from one cluster to another and changing the cluster membership to the one that minimizes the SSE. This is, if ||*x*−*m*_*j*_||^2^ < ||*x*−*m*_*i*_||^2^, data element *x* will belong to cluster *m*_*j*_ instead of cluster *m*_*i*_. *C*-means performs an accurate segmentation of the breast tissues in fatty and dense clusters [16, 23].

### 2.3. Biomechanical Model

Finite element methods (FEMs) are the most popular methods for biomechanical modeling of the soft tissues due to their capability to model irregular structures and complex boundary conditions. In this paper, FEMs are used to perform the simulation of the breast compression during an X-Ray mammography. These simulations were carried out in the commercial package ANSYS. To generate the 3D volume of the breast and the meshes of the three tissues used by ANSYS, the commercial software Simpleware v4.2 was used. The steps followed in this software were as follows.

**(i) Volume Reconstruction**

The 3D volume of the breast was reconstructed from the all segmented tissues from the segmented DICOM image imported by Simpleware.

**(ii) Smoothness**

The smoothing recursive Gaussian filter with sigma values between 0.4 and 0.7 in the *X*, *Y*, and *Z* directions was used in order to smooth the surfaces of the three tissues, the island removal filter was applied to classify free pixels that still could keep in the breast, and the cavity fill filter was applied to fill possible hollows.

**(iii) Mesh Generation**

An algorithm that allows surface adaptation was used in order to achieve the best mesh optimization, reducing the number of elements. In the case of considering the skin as a small surface of constant thickness, the real skin was removed and replaced by a 2D membrane of uniform thickness which covered all the breast.

(iv) *.ans File Generation

A file with all the information about the meshes of the three tissues was generated in Simpleware. This file can be read by ANSYS. ANSYS loads the meshes and allows the deformation to be simulated.

**Figure 5:**
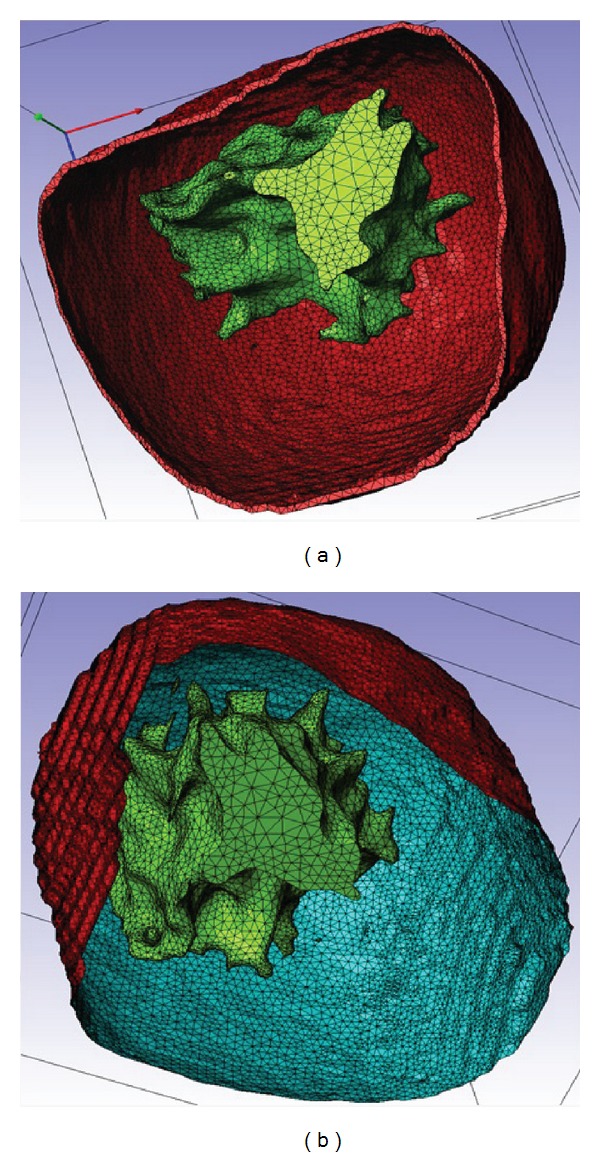
Meshes of the glandular and skin tissues for the two cases under study: (a), considering real skin and, (b), considering the skin as a shell.

Figure 5 shows the meshes of the glandular and skin tissues for the two cases under study: considering real skin on the left and considering the skin as a 2D membrane on the right. The elements chosen to construct the 3D meshes of the three tissues were SOLID186, except when the skin was assumed as a 2D shell. In this case, the chosen element was the shell element SHELL181 with thickness of 1 mm [10, 25].

To simulate the biomechanical behavior of the breast tissues under compression, the hyperelastic model used for the dense and glandular tissues by del Palomar et al. in [10] was also used in this paper. Regarding the skin, the also hyperelastic model proposed by Hendriks et al. for the human skin in [26] was used. For the three cases, the model was a neo-Hookean model, for which the form of the strain energy potential, *W*_NH_, is defined by (3):

(3) $$W_{\text{NH}}=C_{1}\left({\bar{I}}_{1}-3\right)+\frac{1}{d}{\left(J-1\right)}^{2},$$

where *C*_1_ = *μ*_0_/2 and *μ*_0_ is the initial shear modulus of the material, ${\bar{I}}_{1}$ is the first deviatoric strain invariant, *d* is a material incompressibility parameter that is related to the initial bulk modulus *K*_0_ = 2/*d*, and *J* is the determinant of the elastic deformation gradient. The elastic constants used for the three tissues were the constants obtained by these authors in their respective works: *C*_1_ = 3 kPa for the fat tissue, *C*_1_ = 12 kPa for the glandular tissue, and *C*_1_ = 50 kPa for the skin. The values of *d* were obtained using the approximation to incompressible materials.

**Figure 6:**
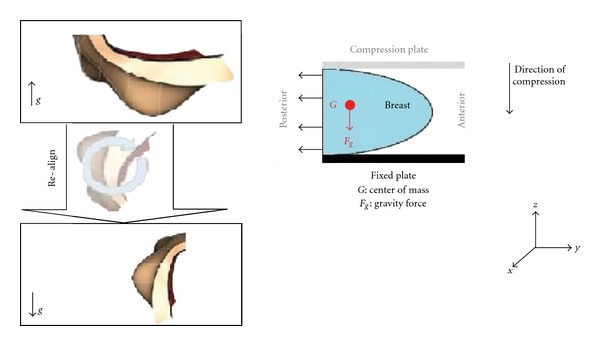
Boundary condition of the problem. Recovering the reference state when the patient is standing up (left) and boundary condition (right).

**Figure 7:**
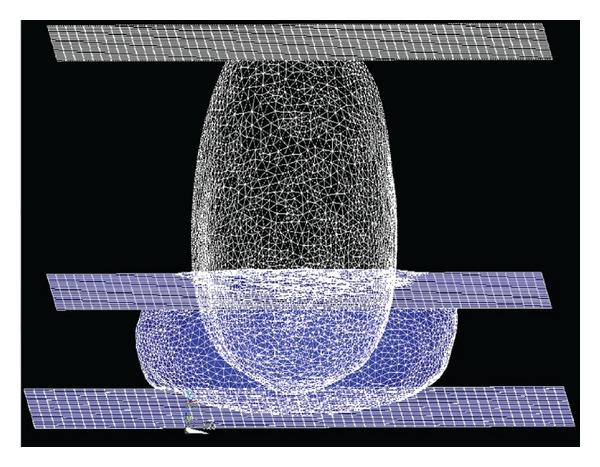
Mammography simulation with ANSYS.

For the simulation of the compression of the breast during the X-Ray mammography, the same boundary conditions were established for both cases, considering real skin and considering the skin as a shell of uniform thickness covering the breast. The gravity was applied twice in order to, first, recover the reference state of the breast when the patient is lying in prone during the MR scanning and, second, establish the real state of the breast when the patient is standing up (Figure 6, left). Regarding the boundary conditions of the problem, the displacement of the nodes that belonged to the chest wall was restricted in the anterior-posterior directions [27] (Figure 6, right).  Figure 7 shows the simulation of the compression in ANSYS.

## 3. Results

### 3.1. Results of the Skin Segmentation Method

Fifteen segmented breast DICOMs were analyzed by three experts (of both hospitals) and compared with a segmentation method that used a fixed skin thickness value of 3 mm to determine skin [17]. This fixed thickness method was rejected by the three experts due to excessive fatty skin tissue in most of the slices (they classified a high amount of slices per case as “Bad,” more than 60.00%). In order to analyze the proposed segmentation, the experts classified each slice in “Bad” (if skin area takes air or is excessively fat), “Tolerable” (if skin is a bit fattier than expected), and “Good.” This validation shows a high percentage of valid slices with a low amount of “Bad” slices (Table 1).

After asking the experts, most of “Tolerable” slices belonged to regions that had experimented skin pixel addition for 3D correction (as explained before). Some slices classified as “Bad” feature air mistaken as skin in air regions naturally formed by patient's position and breasts, and other slices had been classified as “Bad” because skin pixel addition had created skin in slices that did not contain it. However, the percentage of those “Bad” slices is very low when compared with the percentage of the valid ones (Good and Tolerable).

### 3.2. Results of the Simulation of the Breast Compression

To study the influence of considering real skin and considering the skin as a 2D membrane of uniform thickness covering the breast in the simulation of an X-ray mammography, the reaction forces on the plates of the mammograph were obtained and compared with the reaction forces obtained using classical methods that model the skin as a 2D membrane that covers all the breast for seven cases.  Table 2 shows the results. As it can be observed in this table, the committed error when the skin is approximated to a 2D membrane is considerable in most of the cases.

## 4. Discussion

Skin is an important factor that must be taken into account when there is some kind of breast segmentation in MRI. Skin intensity level, similar to dense tissue, is an issue that must be fixed in order to obtain accurate information: breast density analysis or breast segmentation will benefit from a correct skin management [14–17].

The segmentation process described in this paper uses a curvature flow filter to minimize MRI noise and to prepare it for a cluster analysis. *C*-means with 4 clusters is able to differentiate image parts, and two of those parts, which contain skin with some dense tissue, are reanalyzed with a new *C*-means that tries to extract skin pixels. Final improvements as smoothness filter and 3D skin search are justified by the objective of build a biomechanical 3D model to be virtually compressed, so the 3D view must be as complete as possible.

Validation carried out by experts shows a high percentage of valid slices with a low amount of “Bad” slices. And most of those “Bad” slices were classified as “Bad” due to 3D completion or confusion with interbreast air features.

Regarding the influence of considering real skin in the simulation of the breast compression during an X-ray mammography, it has been proved that the approximation of the skin to a 2D membrane for this simulation can provide important errors that can be swept by the algorithms that fuse information of different imaging modalities. This issue has been proved using a neo-Hookean model for the three tissues, model that has been frequently used by other authors, and with not very complicated boundary conditions. Therefore, further studies with more complicated models and boundary conditions should be performed in order to deeply analyze the influence of this kind of approximation. However, the method of skin segmentation presented in this paper opens the possibility to use real skin for the biomechanical models of the breast without the necessity of any approximation for the skin.

## 5. Conclusions and Future Work

In this work, a novel method of accurate skin segmentation has been presented and validated by experts in radiology. Some improvements will be made like, for instance, an intensive search in the nipple region for those MRIs where it is not correctly segmented or computation speed increase. This method will be used to study the impact of considering real skin in biomechanical models of the breast, dense-fatty tissue segmentation without skin interference, and possible impact in the simulation of the load states of the breast in the different imaging modalities.

## Figures and Tables

**Table 1:** Means of validated cases (percentage of slices belonging to each category) by three experts.

Case	Good	Tolerable	Bad
1	61.67%	21.25%	17.08%
2	57.50%	27.92%	14.58%
3	70.43%	21.66%	07.91%
4	62.93%	29.16%	07.91%
5	47.51%	33.33%	19.16%
6	54.17%	27.08%	18.75%
7	62.50%	22.92%	14.58%
8	58.76%	29.58%	11.66%
9	56.68%	27.91%	15.41%
10	60.42%	27.50%	12.08%
11	40.00%	40.84%	19.16%
12	58.76%	27.91%	13.33%
13	52.51%	30.83%	16.66%
14	52.50%	28.33%	19.17%
15	56.25%	25.00%	18.75%

**Table 2:** Results of the simulation of the breast compression.

Case	Breast compression %	Real skin reaction force (*N*)	Skin as 2D membrane reaction force (*N*)	Committed error
1	43.4%	30.745	25.891	15.8%
2	31.8%	246.73	189.44	23.2%
3	29.4%	172.90	129.37	25.2%
4	33.5%	96.938	92.763	4.3%
5	31.9%	155.68	126.13	19.0%
6	30.1%	45.653	41.435	9.2%
7	41.1%	54.858	46.273	15.6%
